# The impacts of learning analytics and A/B testing research: a case study in differential scientometrics

**DOI:** 10.1186/s40594-022-00330-6

**Published:** 2022-02-14

**Authors:** Ryan S. Baker, Nidhi Nasiar, Weiyi Gong, Chelsea Porter

**Affiliations:** Graduate School of Education, University of Pennsylvania, Philadelphia, PA 19104 USA

**Keywords:** Scientometrics, A/B testing, Learning analytics, Online learning, STEM education platform

## Abstract

**Background:**

In recent years, research on online learning platforms has exploded in quantity. More and more researchers are using these platforms to conduct A/B tests on the impact of different designs, and multiple scientific communities have emerged around studying the big data becoming available from these platforms. However, it is not yet fully understood how each type of research influences future scientific discourse within the broader field. To address this gap, this paper presents the first scientometric study on how researchers build on the contributions of these two types of online learning platform research (particularly in STEM education). We selected a pair of papers (one using A/B testing, the other conducting learning analytics (LA), on platform data of an online STEM education platform), published in the same year, by the same research group, at the same conference. We then analyzed each of the papers that cited these two papers, coding from the paper text (with inter-rater reliability checks) the reason for each citation made.

**Results:**

After statistically comparing the frequency of each category of citation between papers, we found that the A/B test paper was self-cited more and that citing papers built on its work directly more frequently, whereas the LA paper was more often cited without discussion.

**Conclusions:**

Hence, the A/B test paper appeared to have had a larger impact on future work than the learning analytics (LA) paper, even though the LA paper had a higher count of total citations with a lower degree of self-citation. This paper also established a novel method for understanding how different types of research make different contributions in learning analytics, and the broader online learning research space of STEM education.

## Introduction

### Online learning platforms

In just a few short years, large-scale online learning platforms have gone from a rarity in education to commonplace. From K-12 to higher education (HE) to post-HE workforce learning, increasing amounts of education take place partially or fully online via these systems. Even before the shutdowns and quarantines of 2020 forced entire school systems and universities to go fully online, the use of online learning platforms was expanding rapidly. Especially in STEM education, interactive learning platform usage has been rising for years. Thus, it is reasonable to hypothesize that many of the schools, universities, instructors, and students who switched to much heavier use of online platforms—and have now become accustomed to their usage and benefits—will continue to use these platforms going forward.

Online learning platforms provide several benefits for learners. First of all, online learning platforms substantially increase the feasibility of access (Park & Choi, 2009)—a benefit first to non-traditional students unable to come to campus at specific hours, and now to the millions of learners for whom coming to a campus poses a risk of life-threatening infection. Furthermore, while not all online learning platforms provide better learning than traditional instruction (and some provide worse learning), heavily research-based platforms such as intelligent tutoring systems can benefit learning substantially. A meta-analysis by VanLehn (2011) suggests that, on average, intelligent tutoring systems (ITS) improve learning by 0.76 standard deviations compared to traditional curricula. ITS have been particularly useful in enhancing STEM education (Fletcher, 2018; Graesser et al., 2018), and STEM learning has continued to be a primary focus of ITS research over the years (Guo et al., 2021). Even more straightforward computer-aided instruction environments still produce benefits for learning (VanLehn, 2011). Online learning environments also facilitate providing actionable information to instructors, information that can be used to drive beneficial interventions, both in K-12 and higher education (Valle et al., 2021; Verbert et al., 2013).

For these reasons alone, online learning has made a positive impact. However, there is another major benefit of online learning: facilitation of basic research on learning (Stamper et al., 2012). Broadly, there have been two primary uses of online learning platforms to support research—making it easier to conduct experimental (or quasi-experimental) research on learning design, and the availability of data that makes secondary analyses possible. This benefit has been realized in concrete improvements to online STEM education platforms (Inventado et al., 2018; Mulqueeny et al., 2015; Nye et al., 2018).

Online learning platforms have supported an increasing number of automated experiments. In the early years of the field, single research groups used their own platforms for research (Beck et al., 1999; Mostow et al., 2003). In the first decade of this millennium, large-scale initiatives such as the Pittsburgh Science of Learning Center DataShop created an infrastructure that allowed hundreds of studies to take place in classrooms, enabling the creation of a theoretical framework on when specific learning designs were appropriate (Koedinger et al., 2012, 2013). However, each study took considerable effort and resources to realize in classrooms. More recently, the ASSISTments platform (Feng et al., 2009; Heffernan & Heffernan, 2014), a STEM education platform focused on math, has created an infrastructure that enables automatic deployment of studies across the web, supporting dozens of studies by external researchers in the thousands of mathematics classrooms using their platform across the USA (Ostrow & Heffernan, 2019). It enables researchers to conduct A/B tests using its E-TRIALS architecture (previously called the ASSISTments Testbed), thereby simplifying the process of creating and conducting studies on the platform. ASSISTments has historically been used both for in-class blended learning and homework, with the proportion using it for homework increasing over time. Prior to COVID-19, the ASSISTments user base averaged around 50,000 students a year, but has increased tenfold (to over half a million) since the beginning of the pandemic. MOOC platforms have also increasingly supported researchers in conducting A/B tests and other types of studies (Reich, 2015). In one case, a group of researchers tested a specific intervention across 247 courses taken by millions of learners (Kizilcec, 2020).

The use of online learning platform data in secondary learning analytics (LA) analyses has also exploded in recent years. Data sets from the Pittsburgh Science of Learning Center DataShop (Koedinger et al., 2010) underpinned the earlier years of research in educational data mining, with 14% of analyses in early years using DataShop data (Baker & Yacef, 2009). Since then, a variety of other learning platforms have shared their data, either publicly or with smaller numbers of selected colleagues. Platforms such as edX, Coursera, and ASSISTments have had their data used by dozens of researchers. Specific data sets have become the standard for comparison of algorithms across papers—for instance, many recent papers have used a specific public data set from the math platform, ASSISTments, to study student knowledge modeling (Khajah et al., 2016; Yeung & Yeung, 2018; Zhang et al., 2017).

However, it is not yet fully understood how these two scientific uses of learning platforms (A/B testing and LA analyses) impact the scientific community, especially in STEM education. There are several potential impacts—for example, the availability of these platforms opens up research to a broader community of scientists, facilitating research and making it less expensive to conduct. In this paper, we asked the question, how does the research these platforms afford impact scientists beyond the ones who specifically conduct these two types of studies using the platform. How does a learning platform impact educational research more broadly?

### Scientometrics on learning analytics

To answer this question, we looked at ideas from scientometrics, the field of scientific study which studies the properties of science itself, by studying the properties of scientific publication.

In recent years, scientometric research has become popular in a variety of areas of educational research, particularly in learning analytics, one of the two applications of large-scale online STEM learning environments that this paper will investigate. Learning analytics researchers have used scientometrics to answer a wide range of questions. However, generally, these questions have been about understanding the current state and research topics of that specific research community, rather than more in-depth questions about the impact of different types of scientific research within the field.

For instance, researchers have analyzed which papers are most cited in scientometrics in learning analytics (Baker & Yacef, 2009; Dawson et al., 2014; Waheed et al., 2018), and have ranked universities and scholars in terms of their quantity of research output and collaboration (Fazeli et al., 2013; Nawaz, et al., 2013; Waheed et al., 2018). However, while these types of papers help to establish the current landscape of that research area, they do not necessarily expand our knowledge of how the online learning field is making progress scientifically through using learning analytics.

A second type of scientometric research in learning analytics has connected to the question of whether current research is equitable. In this vein, there have been multiple analyses of how nationally, racially, and ethnically diverse researchers in the field are (Chen et al., 2020; Maturana et al., 2013; Nawaz, et al., 2013; Waheed et al., 2018). Research has also investigated how nationally, racially, and ethnically diverse the learners in learning analytics research are (Paquette et al., 2020)—this research found that most papers do not even mention the background of learners, making it difficult to evaluate whether our field is paying attention to whether our findings work across populations.

A third category of scientometric research focused on learning analytics has investigated the topics being studied. Researchers have investigated how the topics studied in learning analytics shift over time (Derntl et al., 2013), how the topics studied differ between learning analytics and educational data mining (Chen et al., 2020; Dormezil et al., 2019), and the relationships between the topics published on (Zouaq et al., 2013). Relatedly, researchers have also analyzed the collaborations between researchers with different disciplinary backgrounds (Dawson et al., 2014).

### Scientometrics on why papers are cited

As the previous section indicates, scientometrics has been fairly prominent within the learning analytics community. However, relatively little work has looked at why learning analytics papers are cited, or whether the different types of learning analytics research conducted leads to different citation patterns, in a deeper fashion than just comparing citation numbers between broad comparisons, such as asking whether learning analytics or educational data mining papers are cited more often (Chen et al., 2020; Dormezil et al., 2019). We know from the large numbers of citations that learning analytics research is making a scientific impact, but the question remains how that impact is being made. How are the papers impacting research going forward? Also, considering the issue of online learning more broadly—do different types of papers make different impacts on later research?

The broader scientometrics community has paid more attention to this type of question. Though early work in scientometrics focused on citation counts (Gross & Gross, 1927; Shockley, 1957) or on analyzing the relative contribution of different scientists (Cole & Cole, 1972), there was soon increasing recognition of the importance of understanding not just whether or how much a paper or scholar is cited, but why. An early list of reasons why a scholar might choose to cite a specific paper is given by Garfield (1965); this list is expanded upon in an extensive review by Bornmann and Daniel (2008), which reviewed and summarized the extensive work occurring over the intervening decades. This exhaustive list was in turn distilled into a manageable coding scheme by Lindgren (2011).

Increasing understanding that papers are cited for many reasons led to the practice of analyzing the context of a citation to understand why a paper is chosen for citation (Cronin, 1984), reviewed again in extensive detail by Bornmann and Daniel (2008). In brief, researchers may choose to cite a paper for a wide variety of reasons, ranging from the scientific (giving credit to a key contribution, refuting a previously published idea) to the political (citing an important figure in the field, citing papers from the conference or journal being submitted to). Concerningly, in one analysis of computer science education, only a few citations actually involved building on the ideas or theoretical contributions of a previous paper (Malmi et al., 2020).

In this paper, we built on this past work to understand why researchers might cite a paper based on the research use of an interactive STEM learning platform. Specifically, we investigated the differences between the reasons why researchers might choose to cite a learning analytics paper versus an automated experimentation (A/B test) paper. We did this by comparing one highly cited paper of each type, choosing two papers using the same STEM learning platform, published in the same year, at the same conference, by many of the same authors. For each of these two papers, we identified the papers that cited it and then studied the reasons behind those citations.

## Methods

### Research context

We investigated this research question in the context of the ASSISTments platform (Ostrow et al., 2019). ASSISTments is a computer-based math learning system used daily by thousands of students in real classrooms (over 50,000 a year) and hosts primarily middle school math content. Since 2003, the ASSISTments system has supported an expanding population of learners, with particular uptake in recent years in Massachusetts, Maine, and North Carolina. Learners using ASSISTments complete mathematics problems, and can receive multi-step hints or scaffolding on demand or after making errors. ASSISTments provides support for mastery learning, where learners continue working on a skill until they demonstrate they can answer correctly three times in a row, and offers spiraling practice or review functionality as well.

Among computer-based STEM education learning systems, ASSISTments offers substantial support for external researchers. Learning analytics and educational data mining researchers are able to work with (as of this writing) 14 publicly available data sets, which offer extensive interaction log data, combined in some cases with additional data such as field observations of student affect or longitudinal student outcomes. Dozens of external learning analytics and educational data mining researchers have used data from the ASSISTments system in secondary analyses.

ASSISTments also offers substantial support for educational psychology and learning sciences researchers, enabling them to specify and implement randomized experiments on a geographically distributed population of learners. Again, many external educational psychology and learning sciences researchers have used the ASSISTments system to conduct experimental research (A/B tests). As such, the large scale use of ASSISTments for both types of research investigated in this paper makes it an appropriate context in which to conduct a study of this nature.

### Articles studied

In this paper, we compared the types of scientific impact achieved by two papers, referred to below as the “target” papers. These two papers were selected because of their substantial similarity except for their research method and topic. While leveraging the two different opportunities for research that ASSISTments affords, the two papers were both published in 2006, and at the exact same scientific conference (Intelligent Tutoring Systems). These two papers even share the same second (and senior) author, controlling to a degree for citation patterns due to author reputation.

The first paper (henceforth referred to as the A/B test paper or paper AB), (Razzaq & Heffernan, 2006), compares two pedagogical strategies within the ASSISTments system, on-demand hints and automated scaffolding, assigning students to receive one condition or the other, and then evaluating the impact on student learning.

The second paper (henceforth referred to as the log analysis paper or paper LA), (Walonoski & Heffernan, 2006), uses log data to develop an automated detector of student disengagement, and then uses that detector to investigate the disengagement behavior seen.

### Obtaining citations

In November 2019, we used Google Scholar to obtain every scientific document citing either of these two articles. An article was considered if the full text could be obtained either openly over the internet, through the University of Pennsylvania library, or through interlibrary loan. Both peer-reviewed and non-peer-reviewed (i.e., dissertations, xArxiv, white papers) documents were considered. Only articles in English were considered within this review. Duplicates were eliminated.

**Fig. 1:**
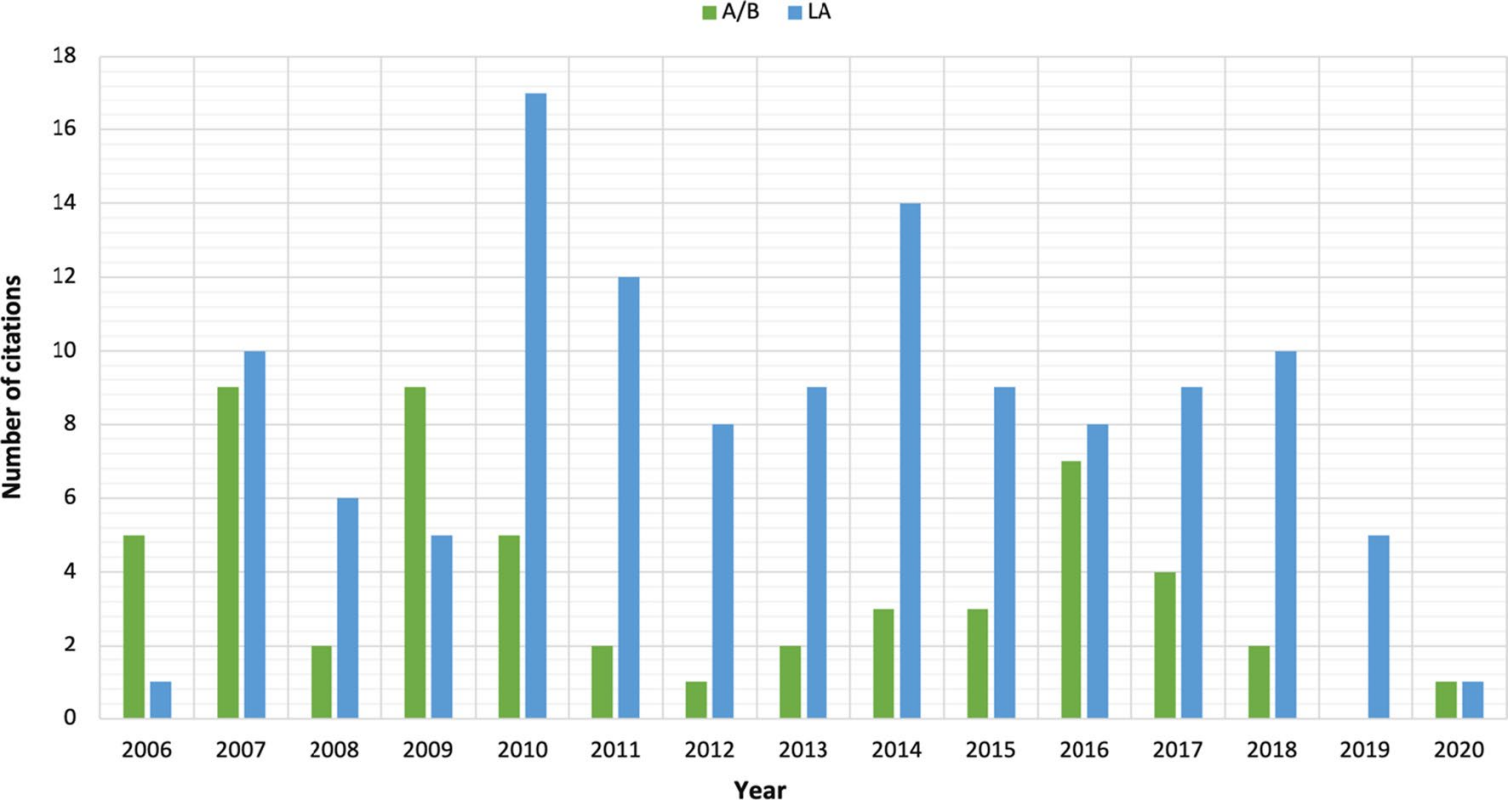
The time series of the citations by year for the two articles

This procedure produced 55 full-text documents citing the AB paper and 159 full-text documents citing the LA paper. The time series of citations spanned from the initial year of publication of the two articles (2006) until the year after the citing articles were collected (2020). This later year was seen because we harvested the papers while they were still in press. The time series, in Fig. 1, shows how the number of citations for these two target papers has changed over time. As the graph shows, the A/B paper was most cited in the first and third years after its publication, waning in popularity in the years after that, but experiencing a second (lower) peak in popularity a decade later. By contrast, the LA paper was slower to reach its peak, reaching its peak in the fourth year after publication, and slowly declining in popularity after that. After 3 years, the A/B paper would have appeared the more influential paper, but beyond that point, the LA paper established a yearly lead in the number of citations that it has maintained until the end of the period of analysis.

The different total number and pattern of citations for each paper may be due to several factors, including the development of these two fields. The learning analytics community (and related scientific communities) have grown rapidly since the start of the educational data mining and learning analytics conferences (in 2008 and 2011), increasing the number of papers that could cite a learning analytics paper, particularly in more recent years. In addition, it is easier to download an existing data set and conduct secondary analysis than to design, conduct, and analyze a study, lowering the barrier for additional researchers to follow-up a study using learning analytics as compared to A/B testing. Each of these factors—as well as other aspects of the scientific and technical merit of the two papers—may have shaped the pattern of citation of these two papers over time.

### Coding scheme

Within each citing article, we identified each of the citations of each target paper. For instance, in some cases, a citing article might cite either the AB paper or the LA paper (or both) multiple times. We then developed a coding scheme for the reasons why a citation might cite an article. The process of developing this coding scheme was led by the third author. Our first step for developing this coding scheme was to take an extensive list of reasons why people cite published articles. We used the list published in Lindgren (2011), which had been distilled from a review of 30 studies on citing behavior (Bornmann & Daniel, 2008). We then eliminated reasons not found in our articles or that would not be explicitly stated in the text around a citation. For instance, Lindgren (2011) notes that authors may cite one paper over another due to the availability of full text for one of the papers—this may be true, but will not be easy to identify from the text around a citation. We then removed or merged categories that we did not feel confident could be differentiated from each other. Finally, based on a quick read-through of the citing papers, we selected and compiled the most relevant citing reasons into our final coding scheme for the two target papers. As will be noted below, not all of the categories we chose to code were ultimately found within the citing papers. The final coding scheme was as follows:

### Publication-dependent reasons

Citation due to some attribute of the publication being cited (the “target” article):

P1: The target paper was the original publication in which an idea or concept was discussed—a “classic” article.
P2: Using/giving credit to ideas, concepts, theories, methodology, and empirical findings by others.
P3: Earlier work on which current work builds.
P4: Providing background reading, to give “completeness” to an introduction or discussion.
P5: Empirical findings that justified the author’s own statements or assumptions.
P6: Refuting or criticizing the work or ideas of others.
P7: Mentions of other work (“see also”, “see for example”, “cf”, “e.g.”, “i.e.”) without further discussion.
P8: Used target paper’s dataset for secondary analysis.

### Author-dependent reasons

Citation due to some attribute of the author being cited (the “target” article):

A1: Paying homage to a pioneer in the research area/giving general credit for related work.
A2: Ceremonial citation, the author of the cited publication is regarded as “authoritative”.
A3: Self-citation: one of the authors was also an author on the target article.

Note that this coding scheme is not exhaustive; some citations may not be coded as representing any of these categories.

A subset of citations in each citing paper was coded in terms of this coding scheme by two coders (either the second and fourth author, or the third and fourth author). If a coder judged that a paper was cited for multiple reasons, multiple codes were given. As a pre-processing step for our final data set, if a citing paper cited a target paper multiple times for the same reason, it was counted a single time—i.e., if the citing paper cited the target paper for reason P1 in three different places, it was treated as a single citation because of reason P1.

The proportion of each citation category found across citing papers was compared between the two target papers (i.e., A/B versus LA) using the Chi-squared test. We discuss the implications of applying different post hoc controls (Bonferroni versus Benjamini–Hochberg) within the results section below.

Statistical power was calculated using G*Power 3.1.9.4, assuming an effect size where one paper was cited 50% more than the other paper for a category occurring half the time in the less frequent paper (risk ratio  =  1.5), with the allocation ratio set to the same ratio as seen for papers AB and LA, and α set to 0.05, using the *Z* test of the significance of the difference between two independent proportions (this test is mathematically equivalent to *χ*^2^ with one degree of freedom—for a given data set, it will obtain the exact same p values). For this test, statistical power of 0.8 would be achieved with samples of 40 and 94, smaller than our current sample.

### Inter-rater reliability

Inter-rater reliability (Cohen’s Kappa) was calculated for each coding category, treating each category as independent since coding was non-exclusive. The average Kappa across categories was 0.740 (not including category A3, for which there was full agreement). Kappa was above 0.6 for every category, placing all categories in the “substantial agreement” or “almost perfect agreement” categories of Landis and Koch’s (1997) guidelines. Categories P1, P6, A1, and A2 were never coded for any citation by either of the two coders. In the case of A1 and A2, this may come from the difficulty in identifying an author-dependent reason for citation from the text of the paper; much of the research on author-dependent reasons for citation has involved self-report rather than content analysis of articles (Vinkler (1987), see review in Bornmann and Daniel (2008)).

## Results

After establishing inter-rater reliability, the fourth coder coded every citation in every paper. We next analyzed the prevalence of each citation category for each paper, and whether the prevalence of any citation category was statistically significantly different for the two types of papers. As mentioned above, within analysis we considered each citing paper and reason combination only once for each target paper, even if a target paper was cited for the same reason more than once in the same citing paper.

Table 1 shows that the most common citation category for both papers, was P2, using/giving credit to ideas, concepts, theories, methodology, and empirical findings by others. It was seen in around half of the citations (averaged at the level of citing papers) for each target paper. Two categories were seen between 20 and 40% of the time for both papers: P4, providing background reading, to give “completeness” to an introduction or discussion, and P7, mentions of other work (“see also”, “see for example”, “cf”, “e.g.”, “i.e.”) without further discussion. Self-citations (A3) were substantially more common for paper AB (35.4%) than paper LA (9.7%). The remaining three categories were seen less than 10% of the time for both papers.

**Table 1:** The prevalence of different citation categories for each of the two paper types

Reason for citation	Average prevalence (paper AB) (%)	Average prevalence (paper LA) (%)	Risk ratio	p-value
P2: Using/giving credit to ideas, concepts, theories, methodology, and empirical findings by others	47.9	56.5	1.18	0.314
P3: Earlier work on which current work builds	**8.3**	**2.4**	**3.46**	**0.078**
P4: Providing background reading, to give “completeness” to an introduction or discussion	25.0	28.2	1.13	0.67
P5: Empirical findings that justified the author’s own statements or assumptions	4.2	2.4	1.75	0.541
P7: Mentions of other work (“see also”, “see for example”, “cf”, “e.g.”, “i.e.”) without further discussion	**20.8**	**35.5**	**1.71**	**0.063**
P8: Used target paper’s dataset for secondary analysis	4.2	2.4	1.75	0.541
A3: Self-citation	**35.4**	**9.7**	**3.56**	** < 0.001**

Statistically significant or marginally significant differences between the two paper types are given in boldface

We then compared the prevalence of each citation category between paper AB and paper LA using a Chi-squared test. This test assumes the paper AB and paper LA are cited by different sets of papers. In practice, only 3 papers cited both of these two papers (out of a total of 214 papers), so this seemed like a safe assumption rather than a situation where a significantly more complex method tailored to partial overlap of data sets would be warranted. All 214 papers were coded and included in the analysis. Our effect size measure, given in Table 1, was a risk ratio, which indicated how much more likely the more common category was than the less common category (1  =  equal proportion; 1.5  =  50% greater proportion for the more common category).

The only category that was fully statistically significant was the self-citation rate. Paper AB was self-cited 35.4% of the time, while paper LA was self-cited 9.7% of the time, *χ*^2^ (1, *N*  =  214)  =  16.35, *p* < 0.001.

Two other categories were marginally statistically significant: P3 and P7. Category P3 represents earlier work on which current work builds. Category P3 was over three times more common for paper AB (8.3%) than paper LA (2.4%), *χ*^2^ (1, *N* = 214) = 3.10, *p* = 0.078. Category P7 represents mentions of other work (cf., e.g., i.e., etc.) without further discussion. Category P7 was more common for paper LA (35.5%) than paper AB (20.8%), *χ*^2^ (1, *N* = 214) = 3.45, *p* = 0.063. The full pattern of statistical evidence is given in Table 1.

Since we ran seven statistical tests, there was an inflated risk of Type I error. One comparison (A3) had *p* < 0.001, so would have been significant even under the highly conservative Bonferroni post hoc adjustment. All other tests were marginally significant or non-significant, and therefore would have been non-significant under an appropriately conservative post hoc adjustment that would attempt to achieve a false discovery rate of 5%, such as (Benjamini & Hochberg, 1995). Therefore, we should consider all apparent findings as tentative; more conclusive evidence on the findings seen here will need to await a substantially larger sample. Given the relative rarity and small frequency of category P3 in specific, statistical power appeared to have been insufficient despite our initial check. Repeating our statistical power check from earlier, we determined that achieving statistical power of 0.8 for a category as rare as this one would require a sample of 583 citing papers. Therefore, a larger number of target papers will need to be analyzed in order to have more conclusive confidence.

## Discussion

In this paper, we investigated the reasons behind why scientists cited the two papers, which used the same STEM education learning platform to do two different kinds of research—automated A/B testing and learning analytics research.

Our findings show that both papers appeared to be cited primarily in terms of publication-based reasons rather than author-based reasons (except for self-citation). However, this may simply reflect the difficulty of identifying author-based reasons for citation. Even if a citation is made solely due to the cited author’s political power, it may still be couched in terms of scientific merit. For instance, some of the category P7 (citations to a paper as an example of some more general category, without further discussion) may actually reflect political/author-based citation. More reliably determining whether some citations are political in nature may require another method of analysis, such as surveying authors anonymously (Vinkler, 1987).

In comparing the two articles, we found only one fully statistically significant difference between the two papers: the A/B testing paper was self-cited over three times as often as the LA paper. Given that both papers involved an overlapping senior author, and almost all of the self-citations involved this senior author rather than the junior authors, this difference is probably not due to differences between the papers in author egos or the desire to self-promote. This is an important check as Lindgren (2011) identified that professional status affects the citation count. Instead, there may have been different reasons for self-citation between the two papers (which did not show up in the formally coded reasons). One possibility is that paper AB built a methodological base that future papers by the same group built on top of. A second possibility is that the paper’s results were more foundational for that group’s later work. A prior study suggests that conceptual papers are currently cited heavily to set the groundwork for this emerging field (Dawson et al., 2014). A third possibility is that the A/B paper was self-cited more often than the LA paper in response to that paper’s lower rate of external citation—the authors saw the paper receiving less attention and attempted to attract attention to it through self-citation; in other words, the same desire to self-promote manifested differently for these two papers. There is some evidence in favor of the second possible explanation coming from the marginally significant difference between the papers in reason P3 (earlier work on which current work builds)—over three times as many citations to paper AB were for reason P3 than in paper LA. Of course, it is uncertain from our limited data set whether this finding would apply to other papers based on A/B testing as well.

By contrast, references to paper LA were marginally significantly more likely (a little under double as likely) to be for reason P7 (mentions of past work without future discussion). This suggests that paper LA may have been seen as an important result by the field, but was not directly built on by future researchers. Again, it is not yet clear whether this finding would apply to other papers using learning analytics methods, or whether it was specific to this result. P7, an author using citations to direct their audience to further reading, has also been cited as one of the most common reasons for citation in computer science research within (Harwood, 2009), which used qualitative interviews to study the functions of citations.

One key limitation to this study is that it investigated only a single matched pair of articles (albeit a closely matched pair). To draw more substantial conclusions, this work must be replicated with a broader range of publications, drawn from a greater variety of publication venues, and learning platforms. By doing so, we can determine if the tentative findings seen here apply across the full spectrum of papers that use STEM learning platforms.

In considering these research findings, it is important to acknowledge the multiple confounding variables which might play a role in these results, but which are not addressed in the current study. For instance, the histories and patterns of usage of the two techniques—A/B testing and Learning Analytics—differ which in turn has led to a different set of researchers being interested in each technique. Various aspects of the training of researchers in each community may lead to papers being cited in different ways. Although the two papers were presented at the same conference in the same year, the two papers were presented in separate sessions—the AB paper was presented in the ‘Scaffolding’ session, and the LA paper was presented in the ‘Gaming Behavior’ session. For the AB paper, no other author who presented in that session cited the target paper. However, for the LA paper, the first three authors of the other two presentations in that session cited the target LA paper (Baker, Corbett, and Koedinger). However, while this could reflect an influence of attending the session, this also might simply reflect that the topic of the LA paper (gaming the system) was a topic that Baker, Corbett, and Koedinger had previously published on and continued to publish on after that point. In other words, these researchers probably would have cited the LA paper regardless of whether they had been in the session. Nonetheless, it cannot be discounted as a possibility that the attendees or other presenters in the session could have influenced the citation patterns of the article going forward. There also could be differences in the expectations of the communities of researchers publishing in each of these areas as well as in the journal editors who select the reviewers who encourage or discourage specific types of citation. These factors might have shaped the patterns we report in this article. Finally, the timeline and feasibility of conducting work of each type, and other structural barriers and opportunities to conduct these two kinds of research vary, and may have had an impact on what research was conducted in ways that could have driven how each paper was cited.

## Conclusion

This article compared two previously published papers carefully matched on several dimensions, to study the differing citation patterns of papers using a STEM learning platform for different types of research. Overall, within the specific case study paper comparison here, an A/B testing paper appears to have had a larger impact on subsequent work than a learning analytics (LA) paper, despite the fact that the LA paper had a greater total citation count and a lower degree of self-citation.

However, as discussed above, our findings remain tentative due to the limited scope of this case study. To draw more substantial conclusions, particularly for rarer citation categories, this work must be replicated with a broader range of publications, drawn from a greater variety of publication venues and learning platforms. Our current work is a first step towards this larger-scale effort—it establishes a coding scheme for publication reasons relevant to the use of learning platforms in research. More importantly, perhaps, this paper starts the process of building a corpus of codes that can be used to train a machine-learned model to recognize each of these publication reasons. There have been initial efforts along these lines in other domains studied by scientometricians (e.g., Athar & Teufel, 2012; Garzone & Mercer, 2000). Developing such a model will have the potential to greatly speed research of this nature—helping us, in the long term, to understand the impact that different research involving STEM learning platforms has on the broader research community studying STEM learning.

## Data Availability

The datasets generated and/or analyzed during the current study are available in the [ASST-citation-comparison-1] repository, https://www.upenn.edu/learninganalytics/ASST-citation-comparison-1/A_B_coding_book-lineup.csv, https://www.upenn.edu/learninganalytics/ASST-citation-comparison-1/EDM-Coding_Book-lineup.csv

## Abbreviations

HE: Higher education
ITS: Intelligent tutoring systems
LA: Learning analytics
STEM: Science, technology, engineering, and mathematics
